# How Do Social Workers Cope with Hopeless Missions and Dirty Work? A Biographical Approach to Class and Gender Variations

**DOI:** 10.1007/s11133-026-09636-1

**Published:** 2026-07-28

**Authors:** Agnès Aubry, Marlène Bouvet, David Pichonnaz, Lucile Quéré

**Affiliations:** 1 University of Applied Sciences and Arts Western Switzerland (HES-SO Valais-Wallis), School of Social Work, Sierre, Switzerland; 2 University of Lausanne, Lausanne, Switzerland; 3 Ecole Normale Supérieure, Lyon, France & Université Grenoble Alpes, Pacte, Grenoble, France; 4 University Paris 1 Panthéon-Sorbonne, Paris, France

**Keywords:** Dirty work, Job satisfaction, Biographical analysis, Socialization theory, Gender, Class, Social mobility, Social work, Adult protection, Legal guardianship, Adult safeguarding

## Abstract

This article looks at the work of legal guardians in Switzerland. They are civil servants, typically qualified social workers, responsible for implementing protective measures (“safeguarding”) for adults considered vulnerable. This work can be understood as “dirty work.” Professionals must provide imposed help to individuals who are often non-consenting. They carry out coercive tasks that are considered morally questionable. Furthermore, guardians interact with stigmatized individuals, which can expose them to the risk of stigma contamination. Finally, they are entrusted with caring for “unplaceable” individuals, those who have often exhausted all other forms of private or institutional support. The article explores how legal guardians cope with this “dirty work.” We distinguish two ideal typical ways of coping, which depend on class and gender socialization, and social mobility patterns. The first coping strategy involves extensively investing in the norms of solicitude, thereby framing guardianship as morally noble work. This approach is primarily adopted by women who have internalized an ethic of care. They engage deeply in relationships with the individuals under their protection, finding personal satisfaction in their work while performing significant emotional labor. The second strategy prioritizes the application of legal norms and the execution of administrative tasks. These professionals, although they can be women, have developed dispositions typically associated with masculinity, such as a positive attitude to coercion. This strategy allows them to legitimize and revalorize their work. By partially avoiding the emotional “dirty work” with service users, they also shield themselves from the various stigmas associated with “undesired” populations.

It’s a job you’re made for or you’re *not* made for. There is no middle ground. Yet, two people can be very different and still be made for it. (Amanda, 31, for two years in post).

## Introduction 

The field of “adult protection,” and more specifically “guardianship” activities, is largely invisible in the sociology of social work, bureaucracy and care (Barnard 2023). Yet, it plays a pivotal role in the landscape of social regulation. In Switzerland, professional legal guardians are civil servants, typically qualified social workers. They oversee the lives of vulnerable adults who are deemed incapable of managing their own affairs, due to mental health difficulties, addictions, or other disabilities, and who are at risk of self-neglect or abuse. These predominantly female professionals (75%) in charge of “protecting” these so-called “wards” must navigate a complex and often contradictory mission of “compulsory care.”

Adult protection authorities deem wards incapable of making decisions in various domains of their lives and issue legal mandates that legal guardians are required to implement. In doing so, they assign social workers the responsibility of deciding on behalf of those under protection, thereby confronting them with significant ethical and moral dilemmas. Some professionals have described it as engendering a sense of embodying the “social police,” exercising a form of “violence” over the adults under their protection. At the front line, they are tasked with taking care of, but also supervising, the wards in various aspects of their lives, particularly their administrative affairs. They are asked by authorities to prevent wards from using their money for purposes deemed inappropriate, prohibitions that can range from a telephone considered too expensive to the purchase of psychotropic substances.

Since this form of protection is often unwanted, professionals are bound to assist individuals against their will. Working with such “involuntary clients” (Trotter 2015) contradicts usual social workers’ aspirations, who “prefer to work with people who clearly choose to be helped” (Hill 2010, 2). This article sheds light on an under-researched profession, which nevertheless exists in most Western countries^1^ and governs the lives of more than 109,000 people in 2024 Switzerland. The association of a helping role with intensive control practices puts these professionals in a paradoxical, in-between position within the state system of social regulation. They embody both the coercive pole – typically represented by police officers – and the compassionate one, associated with social and health workers. As we will see, a number of studies have identified how police officers, and other professionals engaged in activities involving control, deal with the dirty aspects of coercion. Another strand of literature has questioned the dirty work associated with caregiving, as a socially devalued and delegated activity. Our study explores these different aspects of hardship by investigating the research question: *How do legal guardians experience and cope with the dirty aspects of an occupation tainted by coercion, care, and the articulation of the two?*

We propose to explore this question through a biographical approach, allowing us to see variations in the way individuals deal with dirty work, depending on their gender and family socializations, as well as professional aspirations. The workers’ past is often a missing piece of the puzzle in the literature documenting dirty work and coping mechanisms. Previous research tended to focus on the impact of peer and institutional resources, such as work-group culture and professional ideologies, in managing the taints associated with work. This article contributes to the literature on dirty work and taint management strategies by understanding *how legal guardians deal differently with the taints associated with their job, depending on their past*. We will show that the social mechanisms allowing legal guardians to find satisfaction, and even pride and a feeling of success in their work, differ depending on their profile. Literature shows that taint management strategies consist in what could be called “identity work” – how workers “struggle for a favorable sense of self” (Grandy 2008), and reframing strategies aiming at allocating more social value to their work. Although these mechanisms are undoubtedly present in “dirty jobs,” including that of adult guardianship, we would like to dig deeper in workers’ selves to understand how they have internalized a taste for what can be commonly considered distasteful, and how they develop aspirations compatible with working with a stigmatized population and with using coercion.

This study draws on a qualitative research design combining interviews and observations. The article analyzes how guardians exhibit contrasting orientations in their responses to the tainted dimensions of their work. More specifically, we examine the differences between two types of professionals situated at opposite poles of a continuum: on the one hand, those who place care – understood in its broadest sense – at the core of their professional mandate; on the other, those who primarily define their role in legal terms, emphasizing the provision of administrative help. While some professionals adopt intermediate positions, they generally tend to lean toward one pole or the other, depending on their social backgrounds and prior processes of socialization. It is this patterned variation that the article seeks to investigate.

## Literature Review

Viewing adult protection as dirty work helps us to understand the various difficulties that professional legal guardians face in their work. It also helps us to see how a “moral division of labor” (Hughes 1958, 80) tend to delegate necessary but unpleasant tasks to a predominantly female profession. Professional guardianship can be considered a form of dirty work due to the physical, social, and moral taints identified by Hughes (1958), as well as the more recently conceptualized “dirty emotional labor” (McMurray and Ward 2014). In terms of *physical taint*, legal guardians are less frequently in contact with impure materials and danger than other workers (Bouville et al. 2022; Hughes et al. 2017; Seim 2020; Simpson et al. 2014). They may deal with disgust when interacting with wards or visiting their homes. However, they have no obligation to handle physical dirt, in contrast to nurses or domestic servants whose everyday work exposes them to substances considered dirty (Ashforth and Kreiner 1999) – including filth, waste, or bodily fluids.

*Social taints* arise when members of an occupation have frequent contact with stigmatized individuals (Ashforth and Kreiner 1999; Deery et al. 2019; Hughes 1958), which is often the case for social workers in general (Ashforth and Kreiner 2014, 87). The concept is consistent with research findings that have highlighted the “social worth” attributed to patients in healthcare settings (Glaser and Strauss 1964; Hollingshead and Redlich 1958; Roth 1972; Sudnow 1967). These studies have highlighted that professionals are more inclined to assist those who align with dominant social norms. Individuals under the care of legal guardians frequently deviate from the conventional norms of behavior. They often experience multiple forms of stigma, including poverty, mental health challenges, criminal history, disability, and addiction. Consequently, they frequently encounter a lack of “respectability” (Skeggs 1997) and can be seen as undeserving of help. Being delegated the task of taking care of unwanted and undeserving populations, legal guardians therefore face serious risks of “courtesy stigma” (Goffman 1963).

In addition, and contrary to other areas of social work, legal guardians deal with a strong *moral taint* commonly associated with tasks that are considered sinful or dubious, in breach of social norms or ethical standards (Ashforth and Kreiner 1999; Hughes 1958). The coercive dimension of their protective duties, which lies at the heart of their work, gives rise to moral judgements from wards, their families and other social workers who distaste working with involuntary clients. Their prerogatives are often perceived as exaggerated power and they are suspected of being abusive, which can affect legal guardians’ sense of self.

Furthermore, as legal guardians are tasked, within the framework of imposed care, with taking care of individuals facing multifactorial problems, they are also confronted with what has been recently termed as an “emotional taint.” This term refers to the substantial emotional and relational labor demanded of legal guardians in their professional duties (Aubry et al. 2026).

In the following sections, we review the literature on control and care as distinct forms of dirty work, as well as on social theory about biographical approaches.

### The Dirty Work of Law Enforcement and Control

Prior research on workers who use coercive control shows that this “moral taint” (Hughes 1958) might be a severe threat to their identities (Ashforth and Kreiner 2014; Eikenaar 2023; Godin 2008; Tracy et al. 2006). Literature examining the role of police officers underline that their official mandate makes them particularly inclined to act immorally, *i.e*., at odds with dominant social norms. Their use of authority and force can, indeed, easily be seen as an abuse of power (Dick 2005), while the routine exercise of coercive authority does violate “the normal rules of conduct” (Waddington 1999, 299). Research on other law enforcement officers, such as immigration officials, discuss how the daily work of carrying out border control or deportation procedures within a bureaucratic chain of delegation – “getting their hands dirty for others” – involves several moral dilemmas (Borrelli and Lindberg 2018; Fassin et al. 2015). Law enforcement is considered particularly “morally dirty,” and an “inherent suspicion” comes with the use of coercion (Eikenaar 2023, 3). Studies show how frontline law enforcement officials live their job as “necessary evil” (Margolis and Molinsky 2008) and emphasize its legal dimension to counter symbolic contamination (Emirbayer and Johnson 2008; Simpson et al. 2014). Referring to legal frameworks is indeed a way of reframing coercion: by locating authority within “legal-rational” procedures (Weber 1978 [1921]), professionals avoid taking individual moral responsibility for its everyday use (Eikenaar 2023; Kalkman and Kramer 2019).

### Care and Emotional Dirty Work

A substantial corpus of literature demonstrates that care work is frequently delegated, reserved, or assigned to women, on the grounds that they are deemed “naturally” suited to its execution (Gilligan 1977; Glenn 2010; Molinier et al. 2010; Tronto 2020). This assumption is predicated on an androcentric view that deems such work to be devoid of specific skills (Davies 1995). Supporting people experiencing profound and enduring distress requires considerable time (Barnard 2023; Krumer-Nevo et al. 2006, 34) – a resource that professionals often lack. Moreover, the complexity of the problems wards face makes it difficult to provide effective support, and alleviating their suffering is often beyond professionals’ reach (Soulet 2008, 41). In other words, this type of work has every chance of feeding the “precariousness of the meaning of work’ characteristic of ‘dirty work’” (Lhuilier 2005).

Social care work requires a heavy and subtle “emotional labor,” involving displaying the appropriate feelings and modifying one’s spontaneous feelings according to the circumstances (Hochschild 1983). Emotional labor is the ability to “induce or suppress feeling in order to sustain the outward countenance that produces the proper state of mind in others” (Hochschild 1983, 7). Work that demands emotional labor is often considered “low status” because of its association with service occupations that involve serving others (Rivera 2014). More specifically, McMurray and Ward (2014, 1125) have underlined how working “with people who are upset, isolated, suicidal or abusive might be constructed as dealing with an under-conceptualized form of dirt.” Proposing the concept of “emotional dirty work,” they show how emotional labor may have negative social consequences as workers have to deal with “difficult” or “out of place” emotions. Other studies have shown that the embodiment of caring attitudes can also be used as a strategy to cultivate a positive self-image “as humane and sensitive actors” (Kalir 2019, 68) doing “the clean work of caring” (Godin 2008), thus compensating for the moral stigma associated with the controlling aspect of their work.

### A Biographical Approach to Explain Variations in Taint Management Strategies

The variations in dealing with physical, social, moral and emotional taints associated with work benefit from being explored through a biographical approach. Studies about dirty work have tended to homogenize taint management strategies among workers. However, a few emphasize variations, distinguishing among workers showing strong investment and stigma sensitivity *versus* a more detached attitude (Bouville et al. 2022). Attention to these variations is even rarer in research about women workers, although a few studies have examined how conjugal configuration shapes differences in domestic workers’ job satisfaction (Léné 2019). Beyond producing evidence of work practices variations, recent studies have focused on explaining them, not only through organizational factors, but also biographical ones. A relatively recent line of research, in French-speaking countries, has established the impact of past socializations and social trajectories on how work is experienced and performed (Avril et al. 2010; Pichonnaz 2021; Pichonnaz and Toffel 2021). Christelle Avril (2014) has shown that the social and migratory trajectories of support workers significantly shape their (dis)tastes for tasks involving touching the bodies of elderly people. In the specific field of child protection, social workers have been shown to make different decisions according to their family values and their social mobility patterns (Serre 2009). In this article, we tackle biographical factors such as gender and family socializations, as well as professional aspirations to enlighten the varying attitudes of guardians towards dirty work. Acknowledging the effect of social trajectories means shifting the focus on influences workers experienced before entering their occupation and parallel to the workplace. Drawing on Bourdieu’s work and the French tradition of the sociology of socialization (Bourdieu 1984; Darmon 2024; Lahire 2011), we assume that within an organization that imposes a structure, working conditions, and ideology, each worker also brings their own values and ways of doing (Watkins-Hayes 2009). We regard tastes and distastes for tasks as a product of workers’ social histories and past socializations. Our approach is based on the idea that some of the causes of human actions can be reconstructed by identifying (dis)tastes and aspirations. Derived from Pierre Bourdieu’s habitus theory, this approach has *explanatory* ambitions by looking at internalized dispositions. Dispositions “incline agents to act and react in certain ways. [They] generate practices, perceptions and attitudes which are ‘regular’ without being consciously co-ordinated or governed by any rule” (Thompson, J. 1991, 12). To identify dispositions – for example, a tendency to act in line with an ethic of care – it is necessary to observe a series of congruent practices in an individual, in this example marked by acts of compassion. Dispositions or tastes are not deduced from an isolated action or statement, but from recurrences in discourses and practices. We do not claim to identify *all* the causes of certain actions or interpretations of our interviewees. However, we believe that we can demonstrate that some of these actions and interpretations are largely shaped by certain dispositions which originate in specific social experiences, particularly those related to gender and class.

## Methodology

This article draws on qualitative research conducted in three adult safeguarding services in Western Switzerland, referred to as A, B, and C. Although located in three different states (“cantons”), these services are subject to the same federal law, and the professionals perform identical tasks. The data collection process took place from January 2021 to April 2024 and was divided into two phases.

### Interviews and Observations about Work (Phase 1)

The initial phase (January 2021–April 2023) entailed a combination of workplace observations and semi-structured interviews with professional legal guardians. The recruitment process was executed in two stages. Initially, following the acquisition of approval from the respective department heads, we conducted interviews with workers who had previously expressed their interest in participating through responses to an informational email disseminated by management. Subsequent to the initial interviews, ethnographic observations of the interviewees’ work were conducted (see hereinafter), which resulted in the establishment of connections with new professionals who were subsequently invited to participate in interviews. This strategy enabled us to achieve a more diverse array of interviewed workers’ profiles. Openness to participation was notable, as almost no refusals to participate in interviews were received. In this phase, 50 semi-structured interviews were conducted with professional guardians (average duration: 1 hour and 50 minutes) regarding their work. Furthermore, the interviews collected information regarding the participants’ social origins, professional trajectories, and vocational aspirations. In parallel, we conducted 52 observation sessions, each spanning half or a full working day, in which professionals engaged in their routine duties. These observations yielded insights into a wide range of activities related to the management of the social and administrative demands and needs of wards. We observed interactions with wards, as well as computer work and discussions among colleagues and with lower-level managers.

To grasp differences between workers, we have looked at various elements. The way professionals self-identify (*e.g.*, as “social workers” or not) and what they consider is the essence of their work (*e.g.*, “regulations” or “care”) serve as initial indicators. We have also examined the manner in which they talk about their practices, the methods they declare they employ to address specific challenges (*e.g.,* handling repeated requests for financial assistance from wards), the aspects they prioritize when describing working situations, the situations they say create discomfort or negative emotions (*e.g.,* conflicts), and the skills they accentuate (which we were able to validate through observations). To explore the experience of hardship and the strategies used to cope with it, we observed how professionals prioritized tasks, favored certain activities, avoided others and labeled some as unpleasant. We also paid attention to the tone used when communicating with wards, the consideration given to their individual paths and experiences, reactions to situations of aggressiveness, and whether workers tend to engage in or avoid home visits. We have also gathered evidence regarding how workers view wards, including whether they consider them deserving or not, responsible for their life or not, and regarding how they judge emotions and behaviors exhibited by wards. Finally, we have examined the congruence between personal preferences (judgments of situations in private life) and those related to professional activities, particularly in terms of behaviors that are subject to negative evaluation. By gathering information on perceptions of work, judgments, and tastes, we are able not to limit to stated “motives” or “justifications” even though these are important. As Mills argues, “to term them justification is not to deny their efficacy. Anticipations of acceptable justifications often influence conduct” (Mills 1940, 907). This is particularly true of judgments made about wards, which implicitly allow us to study how they are treated.

### Biographical Interviews (Phase 2)

In the second phase (December 2023–April 2024), we conducted ten in-depth biographical interviews (average duration: 2 hours and 30 minutes) to delve into participants’ life events and trajectories. The initial 50 interviews enabled us to establish professional profiles or styles and to reconstruct broad career paths. We employed a purposeful sampling strategy to select ten out of these 50 participants, ensuring a diversity of profiles in the way they see and perform their work as well as in their social backgrounds, previous education and professional experiences, and gendered dispositions. The initial interviews and observations of working practices (phase 1) enabled us to identify dispositions that, while not internalized with the same strength by all workers, appeared repeatedly. These include a compassionate attitude and a tendency to impose oneself or to impose actions upon others. The biographical interviews facilitated the consolidation of the identification of these dispositions while exploring their sociogenesis. Biographical interviews are a valuable tool for reconstructing past socialization contexts and their effects on individuals. This process involves first asking general questions about the past (*e.g.*, “How did things work with your parents?”) and then progressing to more contextualized questions (*e.g.,* “What did your parents do when you brought home a bad grade?”). To assess the socializing force of a past experience and any forms of resistance to it, additional questions were asked about how the respondent reacted to the experiences recounted and the environments frequented, as well as their retrospective judgment of these situations (“Would you do the same with your children?”). This technique places particular emphasis on the pivotal moments and challenges that mark life trajectories, such as the selection of an educational path, the commencement or cessation of employment, leisure activities, the birth of a first child... This type of interview is necessarily long, and they were sometimes conducted in two parts. We insisted that the interviews take place outside the workplace and did everything we could to be received at the respondents’ homes, which was often the case. Conducting an initial interview with the individuals that focused on their work greatly helped us obtain their agreement to a second, biographical interview. Additionally, it made it easier to establish a climate of trust, enabling individuals to discuss topics relating to their past experiences and private lives more readily.

## Findings

Although legal guardians deal with the tainted aspects of their work in different ways, they share a common feeling of being delegated to difficult cases, undesirable people, those who have not been able to be helped by other professionals or institutions. They very often stress that they are “the last link in the chain,” dealing with individuals “nobody wants to look after.” As reported in the interviews, adults under protection can be subjected to pejorative labeling as “welfare cases” or even “fauns” (Sylvie) or “trash” (Eva) by the relatives of social workers themselves.

In contrast to areas of social work that are seen as virtuous and may lead to the empowerment of service users, such as child protection, the situation of people under adult safeguarding measures might never get better. This hardly meets the professional norms inculcated in social work training, which insists on “the empowerment and liberation of people” (IFSW 2014). Another common feeling among legal guardians is, therefore, that they try to “limit the damage” (Juliette) and ensure that the situations of their wards, these “ultra-low threshold people” “don’t get any worse” (Sylvie), or even that: “We just try to avoid shit happening.” (Amanda). In this sense, wards do not represent the “ideal clients” (Becker 1952) of social work. Moreover, professionals must deal with recurring accusations arising from other professionals, wards' relatives, or the general public of not being sufficiently efficient. These elements point to the multiple forms of taint that characterize this work.

In what follows, we focus on two poles among guardians: the compassionate pole and the legal-rational one. Each is underpinned by contrasting attitudes, tastes, and skill sets and is associated with distinct ways of taints management. While intermediate profiles do exist, our analysis concentrates on cases situated closer to one pole or the other, as emphasizing contrasts makes the role of gender and social trajectory more visible. These two poles partly overlap with those identified by Celeste Watkins-Hayes (2009). Drawing on a study of less-qualified welfare caseworkers in the United States working with similar populations as legal guardians, she distinguishes a “social worker” profile – workers who “possess a wide interpretation of the areas in which they should intervene to assist clients” (54) – from that of “efficiency engineers,” whose conception of their role is more narrowly focused on benefit delivery, to the exclusion of other client difficulties. As in our own analysis, Watkins-Hayes concludes that this division reflects “the tension that exists between a counselling role and that of the efficient, rules-observant bureaucrat” (73). In our case, the distinction between the two poles also lies in how legal guardians mobilize compassion and authority in practice. To position a professional closer to the compassionate pole does not imply the absence of coercive practices grounded in institutional authority. Conversely, the exercise of this occupation presupposes a capacity for compassion, even among those situated nearer to the legal-rational pole. What differentiates these poles is therefore not the presence or absence of compassion or coercion, but rather the *frequency* with which they are mobilized and the *forms* they take in practice.

### Compassionate Legal Guardians: From Coercion to Care

A first group of professionals are characterized by having strong compassionate dispositions. These professionals are exclusively women and share the common experience of having taken on a caring role early in their lives – one that has remained uninterrupted. Through these different experiences, they have developed an “ethic of care” (Tronto 2020). This is the case of Eva, who lost her mother at the age of ten. Since then, as the older sister, she has cared for her siblings. As a teenager, supported by her father, she became involved in a religious youth group where she enjoyed “the values of sharing and benevolence,” and discovered the world of volunteering and charities; she also made humanitarian trips to build schools in a deprived area in Asia. As the daughter of a psychologist, she has witnessed her father’s volunteering activities and his habit of working long hours to be available for his patients. Eva feels she has a strong “vocation” for the job of social worker and has never considered any other career since she was 18: “At the end of high school, I decided that I wanted to study social work – for me it was *completely obvious.*” A lot of female professionals with similar backgrounds present their work as “a vocation” and stress their ability to be “there for others” (as Margaux or Nadia put it).

The coping mechanisms these workers use to face social and moral taints are inextricably linked to their past experiences of caring. Embodying compulsory care practices is not a given for these professionals, who define themselves as “sensitive” people, used to “paying attention” to others. As a result, they (re)emphasize, in their discourse but also in their practices, the “social” aspect of adult protection. They regret that this dimension is too often minimized in popular narratives, as one underlines:

Sometimes I think that people misunderstand what we do. [...] I want to be able to provide social care. That’s what drives me. That’s what I do. If I can’t do that anymore, I’m not sure I’ll still find much meaning in social work. (Serena, qualified social worker, 18 years in post, service C)

To make sense of their morally objectionable work, professionals with an ethic of care tend to define themselves as being at the service of others.

#### Guardianship as an Opportunity to “Save” the “Abandoned Ones”

By defining themselves as social workers (rather than “legal guardians,” see hereinafter), guardians from the compassionate pole are particularly attracted to populations often label as “abandoned.” Margaux, for example, is driven by the urge to “save” them – although acting as a “savior” or “rescuer” is not considered appropriate in social work professional norms (Le Bossé 2016). Taking care of those who are “left out” (and can be judged by the general public as not deserving help) is what drives her; she does not experience it as dirty work delegation:

I like these populations that nobody takes care of, that’s it. It attracts me more [than populations targeted by more institutional support, such as migrants]. I also like working in the world of drug addiction, all those areas that are a bit more... Yeah, where I have the impression that they’re being pushed aside. The side that nobody wants to go to. Maybe, sometimes I feel passionate about this kind of thing, like a rescuer – no, never say that, the [social work] school forbids us to say that! (Margaux, qualified social worker, ten years in post, service C)

This interviewee has a long-standing practice of providing support to individuals in need in her private life, for example to her best friend, whose father has been confronted with mental health challenges since she was ten. At her wedding, family members gave speeches about her remarkable capacity to elicit secrets from reticent individuals, as she recalled. Legal guardians such as this one do not mind accomplishing tasks that colleagues would find out of their jurisdiction and would delegate to others, such as support workers: helping wards to shop for clothes, for example. In the excerpt above, it appears that taking care of unwanted or undesirable people is lived as a positive experience and constructed as a form of heroism (producing “saviors”). Those professionals who place helping others at the center of their work and their lives feel that helping those nobody wants to help and that are not easy to help is rewarding: a source of recognition and even pride, not dirty work.

#### Displaying the “Ethic of Care” to Cope with the Moral Stigma

Professionals close to the compassionate pole perform specific “emotional labor” (Hochschild 1983) to be able to listen and make themselves available to wards. They try to alleviate or transform the coercive aspect of the help they provide to “gradually enter into a real partnership” (Nadia). This can be seen in the way some of the female social workers we observed used a compassionate tone when inquiring about the well-being of their recipients during face-to-face interactions, or in the way they take into account their individual paths and experiences, despite having many mandates (Aubry et al. 2026). By acting like this, professionals with a caring profile also try to regulate the emotions of those under their authority (Abstract 1989). Their aim is to transform the negative emotions associated with compulsory care into gratitude, or at least a positive view of the help provided, which requires social workers to invest time in each relationship:

Sometimes I’ve waited a year, trying to build up a relationship with the person first, calling them regularly, going to see them, offering to help, and then all of a sudden there’s an event where you have to be more proactive and the person may realize that it’s actually helpful to have someone who can help them with the paperwork. (Helen, qualified social worker, 15 years in post, service B)

By nurturing relationships with wards and by providing material help, social workers challenge the negative emotions associated with compulsory care and seek to transform what may be perceived as arbitrary and imposed, into support that is timely and responsive to the individual’s needs. By engaging in this practice, they distance themselves from deceptive, intrusive, or confrontational practices and establish a role that aligns with their caring attitudes. The latter can be associated with a slight lack of comfort in the face of conflict, even if it is considered part of the job:

The conflicts... Well, I know they exist, and I am used to them, to being with people who sometimes don’t understand everything [about my limited resources], to being in these situations where I say, “that’s how it works,” “I’m sorry, but I can’t see you now.” I don’t necessarily feel very comfortable with it, but that’s the way it is. (Aline, qualified social worker, 21 years in post, half in service C and in service A)

The biographical interview conducted with Aline clearly shows that she has internalized an ethic of care, which she recognizes herself:

It’s true that when my parents describe me as a child, I was always very much in touch with others, and also very caring, so I think I already had a bit of that Samaritan [*laughs*].

Her past and her present attitude contrast with that of legal-rational guardians who can decline wards requests with minimal difficulty. Another distinction pertains to the frequency of home visits by social workers. Those closer to the compassionate pole undertake such visits more frequently, viewing them as an opportunity to demonstrate their concern for the well-being of the wards and as a means of fostering a more balanced dynamic in the caregiving relationship. For example, Eva justifies her home visits by reframing them as a way of adding a new dimension to relationships, as the wards and her would “not share the same things” in her office, and by insisting on the “pleasure” that some of her wards have had in offering her a cup of tea when receiving her at home. Home visits are an activity that other guardians prefer to avoid, notably because of the risk of physical dirt exposure (see hereinafter).

#### Emotional Dirty Work: Patience and Ability to Reduce Anger

Compassionate legal guardians often stress their will and ability to be committed to their wards. They put an emphasis on their “patience” and their ability to listen to “always the same stories” from wards:

Some people tell me about their stories... Every time she comes, a lady tells me all about her medical issues and doctors, the impact of health care on her life. And for the five years I’ve been following her, it’s always the same stories, it goes around in circles, she goes back to 20 years ago and everything. And for me, it’s OK to just listen to her for one hour, telling me about it. The interns sometimes tell me, “But I just can’t take these stories anymore! It’s always the same thing!” (Eva, qualified social worker, nine years in post, service C)

Legal guardians also need to develop strategies to face sadness, mutism, and threatening behaviors. Their job involves conversations that follow a traditional script, but also “*fausses notes*” (Goffman 1963) or “emotions out-of-place” (McMurray and Ward 2014). They also produce an “objectivation effort” (Bouvet 2023), *i.e.,* gathering information about the main parameters of the person’s life, regardless of their emotional state. This might involve being insulted or shouted at, as shown in the following scene one of us observed:

Mr Z. is in his forties, wears a cap over his messy hair, old clothes, and smells of alcohol. He has come to adult protection service to get some money, and Leah takes the opportunity to inquire about his mental health state. He states that he is “in a bad mood” and raises his voice. He asserts in an aggressive tone that he has only come here to get “money to eat” and does not feel like talking. Leah, keeping her composure, responds, “OK, Mr Z. It’s not a good day then.” He replies in the same tone of voice, “NO, it’s not a good day!” and starts shouting that he never sees her because she is “always on holiday.” Leah replies in a quiet and composed voice, “I was away for seven days last week, Mr Z. I wouldn’t say I’m always on holiday, but I’m available if you want to talk now. You’re not feeling well, are you?” He shouts, “No, I’m not!” Leah looks worried and asks him if he wants to talk about what is upsetting him; he declines. He goes to the counter to get his money and continues to reprimand Leah. She remains calm and invites him to come back at another time. (observation with Leah, qualified social worker, two years in post, service C)

Remaining calm in the face of someone who shouts, moves relentlessly while talking, or remains completely silent, is synonymous with a great deal of emotional labor. Compassionate legal guardians have developed a special alertness to the needs of others during their lives *before* entering social work and *during* their professional careers. These professionals consider it as fully part of their job, and their biographical background helps them to deal with dirty emotional labor. They display a taste for it and even pride themselves on being able to deal with it whereas interns encounter difficulties in doing so, as stressed by Eva above.

### Legal-Rational Guardians: “We Are *Not* Social Workers”

Other legal guardians adopt a different approach, focusing more on the legal and administrative aspects of their work. Although legal guardianship is officially considered as social work and some of the “legal-rational” professionals are qualified social workers, those with such a profile do not identify with the profession: they prefer to be called “legal guardians” or “protective mandates officers.” They conduct less face-to-face encounters with their wards and attach a greater importance to keeping files “tidy.” These guardians present themselves as “stricter,” especially in terms of allowing money for extra spendings, and are more comfortable with conflicts. For Lorraine, a social sciences graduate who has been in service A for two years, facing resistance from wards is common: some “never accept” the intervention. She views the “wrestling match” dynamic with certain wards as part of the process and not an issue. Carine has very similar views:

And then, well... They don’t agree with being under guardianship... We’re in a bit of a permanent tug of war and I think we must accept this situation. Well, that’s the way it is, we give what help we can and then, if they’re not happy, that’s just the way it is. (Carine, qualified social worker, eight years in post, service A)

Carine, who aspires to work in law enforcement, distinguishes her work from that of a social worker: “We’re more into pure budget management [...], setting limits, establishing a framework.” There are several indications that this way of dealing with conflict was internalized *before* entering the profession. Carine describes her upbringing as “strict,” with a rather authoritarian father who was firm on discipline. She never questions this conduct, on the contrary: she affirms her admiration for her father. She also states that she is “easily annoyed” and that “it shows” when she is. She grew up in a family with a comfortable income and never faced the necessity to take care of a dependent person in her private life. Although they do care for their wards, in accordance with the norms of their profession (Aubry et al. 2026), professionals closer to the legal-rational pole have not internalized the same ethic of care as their compassionate colleagues. This is evident in their reluctance to soften the rigid boundaries of compulsory care. What motivates guardians with such a profile is above all the application of “judicial orders,” as in the case of Greg, who aspired to a career in academia but did not manage to pursue it. He was introduced to the profession by a friend who described him as “having all the qualities for it,” as he’s “very precise, square, organized.” These profiles of workers see compulsory care as a “challenge” and have more often worked in prisons. This is the case of Eloise, a criminology graduate who came into adult safeguarding “by accident,” as she puts it, having applied for what she describes as a “dream job” as a prison “assessment officer” but not getting it.

#### A Taste for Highly Deviant Behaviors and the Need to Suspend Internalized Dispositions

These professionals manage the challenges associated with their profession in a distinctly different manner from their “caring” counterparts. A notable aspect of this is their keen interest in highly stigmatized populations, which they often associate with noble work. This association is rooted in the triggering of interest, and at times, a fascination, for behaviors that deviate significantly from dominant social norms, as Carine illustrates:

I like things a bit... I don’t want to say creepy, but situations that are a little out of the ordinary... (Carine, qualified social worker, eight years in post, service A)

Justifying her decision not to study psychology, Eloise explains that she could not see herself listening to people’s “basic problems,” such as “feeling down” or “problems at work.” She therefore opted for a degree in criminology, after having attended a course on prisons as an auditor:

[This course] brought together everything I liked: the somewhat marginal aspect – prison is a very special environment – with disorders, or at any rate with psychiatric or psychological outlooks that are somewhat different. Well, not everyone becomes a delinquent either, we can all become one, but there are stages, you can see people’s paths that are a bit broken, that’s it, I was very interested in that. (Eloise, criminology graduate, 11 years in post, service A)

Confrontation with extreme poverty and deviance is therefore not seen as dirty work; on the contrary, it is a source of satisfaction. It goes hand in hand with a taste for stories and narration: Eloise is inexhaustible in her accounts of work situations that have confronted her with atypical behavior, eccentric reactions or sordid details. This relationship with deviances produces a feeling of singularity (see, about the police: Pichonnaz 2021), which functions as a way of reframing “dirty work” as a noble task, worthy of interest. Eloise explains how, with her colleagues: “Sometimes we tell each other anecdotes and say ‘who the hell [else] does that in their job?’” This statement clearly indicates that working with socially stigmatized populations is a source of pride.

A legal guardian who has recently left the profession talks about the feeling of great satisfaction she gets when, in front of her new colleagues, she can display her habit of being confronted with major deviance. Noting the “silly things” done by her current service-users, she explains that her new colleagues are “a little outraged, shocked,” but that she “temporizes,” explaining that nothing shocks her: “I play down what is brought to me as dramas” (Amanda, qualified social worker, service A). While guardians with this profile like to tell stories of wacky situations, they do not build up a heroic mission that would insist on the physical taint of dirty work. When recounting the situation of a person living with a “hoarding disorder,” for example, Carine emphasizes the atypical nature of the behavior (the person is forced to sleep in a tent in the garden because his house is so cluttered), not the dirtiness inside the home.

In order not to experience confrontation with these populations as dirty work, it may be necessary for guardians to put previously internalized dispositions on hold – what they sometimes refer to as “playing with one’s values.” The ability to suspend judgment is even sometimes presented as an indispensable professional skill, as the following excerpt from field notes shows:

About a man under guardianship, Delphine (art school graduate) says to me [researcher], pretending she’s talking to him, “You don’t work, and you use [narcotics]? That’s not a problem, really.” Eloise, present in the office, laughs, and agrees, jokingly imitating a guardian who would say, “I refuse [to take over] the guardianship of people who don’t work, hahaha.” (Observations, service A)

Eloise then mentions the case of a guardian who only stayed in the service for one year because he thought that the wards needed to get to work, to “get off their butt.” She adds: “We can’t think like that here.” Delphine adds that it’s a job of “compromise”: “You have to play with your values a lot. There are small victories, but many difficulties. There are a lot of things we do [that we put in place], but it doesn’t work. You have to accept that we’re not going to save everyone.” We can see here how differently these guardians think of their roles compared to those closer to the compassionate pole.

The mechanism mentioned by Delphine can be interpreted as a way of dealing with socially stigmatized populations, so as not to experience it as “dirty work.” This is achieved at the cost of putting on hold the dispositions inherited from previous socializations which would incline them to judge wards as lacking merit (because they do not work and use drugs). Another guardian, Carine, says she is “not patient” in her life outside work and easily prone to “irritation.” When asked in an interview to give an example, she recalls a recent party she was invited to as well as solicitations from people begging for money in the streets:

[At the party], there’s this girl but... I think she’s got, like, a mental disorder. During the evening, I had trouble... talking to her. Because, first, she had NO notion of distance, and in fact she talks to me like that [very close] even though we don’t know each other, and then she touches my hair, and in fact, I’m not a tactile person. So I was like “Aaaah, hello...” [*mimics a tensed tone*]. And then every time she came over during the evening, it wasn’t really appropriate. I’m already coping with this all day, I don’t really want to spend my evening being nice and understanding. I actually want to have a beer with people I can have a cool conversation with. And it’s true that working in the social sector makes me... more on edge, in fact, for situations where people come up to me on the street and say: “You got two bucks?” NO, I don’t. And it goes a lot quicker to make me tense. And then, sometimes I’m able to say to myself, “It’s not his fault, let’s relax,” but yeah, I have a lot less patience when it comes to socializing in my personal life. (Carine, qualified social worker, eight years in post, service A)

This excerpt clearly shows that, in her private life, Carine tends not to appreciate behavior that challenges her definition of normality. The fact that she mentions having to “do this all day already” shows that the work context requires her to put this disposition on hold. In addition, Carine mentions that the person she met at the party potentially has a “mental disorder,” but this does not trigger any compassion. In her job, Carine’s patience depends on a moral judgment of non-responsibility concerning wards (“It’s not their fault”), like another guardian, Amanda, who, concerning highly inappropriate sexual remarks made by a ward to a woman, says: “It’s totally inappropriate, but it’s not him talking, it’s the disease.”

We can see how suspending one’s disposition – “inhibiting” it (Lahire 2011) – consists in accepting behavior considered unpleasant or scandalous in private life, with the result being a lack of annoyance towards these behaviors. This ability to put a disposition on hold, which is specific to guardians who have not internalized a compassionate disposition, appears to be fragile: Carine has no problem admitting that she can get annoyed at work and “yell” at wards when she holds them responsible for their behavior.

#### A Taste for Coercion and the Importance of the Legal Framework as Legitimizing Device

Legal guardians close to the “legal-rational” pole easily accept and even claim that the help and assistance they provide is imposed on their recipients. They are also unfazed by, and even display a predilection for, the conflicts that arise from this situation, regarding them as a “challenging” aspect of their work. They regard wards as necessitating oversight and administration in accordance with the legal framework of adult protection, with one individual even comparing his job to that of a lawyer:

I would say that, in a way, lawyers do the same thing [than us]. We’re not lawyers at all but in the end there are articles of law that are quite rigid [...]. We don’t do what lawyers do, but we do bring the particular case into play [...] and we apply it to certain rigid standards. (Greg, social sciences graduate, 13 years in post, service A)

This guardian is one with the strongest distancing discourse toward social workers:

We are guardians, not social workers [...] Being labelled so is a dealbreaker. I didn’t study with the goal of becoming a social worker or an educator. And besides, I find that it’s a category that comes with a lot of connotations.

*What are the connotations?*

It would be something like having a heart of gold, playing the Good Samaritan, basically just floundering around without doing a real job. [Showing] empathy, listening, things that are necessary, and which are part of all jobs I would say, but which belong more to the field of social work [than of guardianship]. [...] The main thing that sets us apart from social workers is that we work under a legal order. In fact, we have a mandate of justice, which is also why, on the salary scale [we are better paid]. (Greg, social sciences graduate, 13 years in post, service A)

For Greg, working in a profession closely related to legal procedures and rules seems to compensate for his “frustration” at working in social services, which seems to be degrading to him. He understates the skills required in social professions and rejects the caring ethics they presuppose. The dissociation from social work – traditionally considered a female domain (Freedberg 1993) – allows him to compensate for the downgrading of his social aspirations (he wanted to pursue an academic career) and to embody hegemonic masculinity scripts associated with rationality, toughness, power, control, assertiveness, or restricted emotions (Canham 2009). The ability to recast his profession as closely linked to the “legal order” allows him to take a symbolically meaningful position. For legal guardians embodying this model of masculinity, coercion is not considered as morally dubious and needing to be alleviated. On the contrary, it serves as a basis for valorization: the legal norms and judicial decisions behind it are a source of social worth. Female workers who, like Greg, do not associate the use of coercion with a moral stigma also distance themselves from being social workers, and thus, from socially hegemonic feminine norms and roles associated with caring practices:

I have colleagues who are much more social, much more involved in helping people and so on. And they love it! Accompanying recipients in daily activities or small acts of daily life [...] Fine, but it actually does us a disservice, because adults under protection say to us [*heavy voice of reproach*] “Yes, but your colleague did it!” And we have had that many times. “Yes, but your colleague did that!” “Sorry, but... Not me!” (Carine, qualified social worker, eight years in post, service A)

These professionals reject qualities and skills that some of their caring counterparts are proud to embody. They refuse to play the role of helper who would personally engage with the adult in their care, preferring to externalize these tasks to others. They tend to not take responsibility for their wards, considering them to be “adults.” For example, Eloise talks about a ward needing help to move flat:

We’re not here to make up for everything, we’re not saviors. We don’t do miracles, we don’t have magic solutions. [I told this ward:] “So if you were on your own, well, you’d do the [apartment] move yourself. So we can find funding for certain things, but if you’re physically capable of doing a move...” Well, that’s up to them. (Eloise, criminology graduate, 11 years in post, service A)

While these professionals better accept the coercive aspect of their job, it can still be seen as morally dubious by outsiders. Thus, although they do not attempt to alleviate the coercive aspect of their job, they stress the legal framework that stands behind their practices to legitimize them:

Social workers [not working in statutory contexts] can give advice, [...] can say: “Well, If I were you….” [With] no judicial power, no legal power, so it’s like: “I gave you the advice, and it’s up to you.” [In legal guardianship] we don’t give [wards] advice, we’re happy to discuss things, we’re happy to negotiate certain things. But there is a point when they sometimes say to me: “Yeah, but it’s not up to you to decide.” Well, actually it is, and I’m telling you that we’re going to do it this way. I always give the reason why we’re going to do it this way. But I’m not going to change my mind and [I say:] “If you don’t agree, you can complain to the Adult Protection Authorities.” And I’ve already had to justify my decisions to the authorities, to justify them in letters and then explain why I’ve proposed it this or that way. I don’t like that term, but it’s true, we have the power to say no and to decide certain things, within a legal framework of course. Within a framework where I have to be able to justify my choices and not fall into an abuse of power. (Eloise, criminology graduate, 11 years in post, service A)

For this group of professionals, a central coping mechanism appears to be the reliance on the adult protection legal framework. The fact that these workers are legally entrusted with the use of coercion seems to diminish the “dubious virtue” attached to it.

## Discussion

In this paper, we analyze a middle-class service job, drawing on Hughes’ work but bringing out key dimensions of guardianship while redefining the contours of “dirty work” theory. By analyzing the well-documented taints – physical, social and moral – experienced by these workers (Ashforth and Kreiner 2014), and by raising the question of an emotional taint, this paper provides valuable insights into the hardships and satisfactions of a job primarily occupied by women. They are the primary responders to pressing social issues such as aging, addictions, mental health problems, and poverty.

### Tainted Emotional Labor

Most of the literature drawing on the concept of dirty work focuses on male working-class jobs, stressing the physical, social, and moral dimensions of stigma (Hughes et al. 2017). Few previous works have applied the same concept to female jobs, grasping hardship and delegation processes in healthcare professions (Molinier 2020; Molinier et al. 2010). We combine these two approaches.

Standing at the crossroads of law-enforcement and social work (Barnard 2023), guardianship entails heterogenous hardships. Like any profession specializing in care, guardians face an excessive workload, low recognition and exhausting emotional demands. Their emotional labor is naturalized and associated with the realm of women’s work, contributing to its invisibility, underestimation, and delegation (Hochschild 1983; Husso and Hirvonen 2012; Tracy et al. 2006). However, Hughes’ concept enables us to demonstrate that this emotional hardship is exacerbated by social and moral issues. Following Rivera (2014), we argue that the analysis of “social administrative professions” has much to gain from integrating these taints to catch the very nature of “emotional dirty work” (McMurray and Ward 2014).

In this light, guardians’ emotional dirty work is multifaceted. The experience of repeated failure in attempts to assist people who are hard to help can lead to feelings of inefficiency or discouragement, which can further compound the emotional burden. Dealing with marginalized populations involves being constantly faced with emotions that are “out-of-place”: anger, extreme sadness, loneliness, atypical and/or strong emotional outbursts, which can “contaminate the clean rational logic of efficiency” (McMurray and Ward 2014, 1128). This emotional labor is intensified, firstly, by the *social taint*. Caring efforts are tainted because they regard individuals of low status who are perceived as undeserving, including by legal guardians’ relatives. Therefore, professionals must deploy cognitive and emotional efforts to resist the public perception of them being of service to “welfare cases.” Some of them also struggle with their own disapproval or disgust towards the wards, which contradicts the social work ethos. Secondly, we distinguish an emotional labor contaminated by the *moral taint*, associated with the use of coercion. As in law enforcement professions, imposed help often leads wards to be unsatisfied or angry due to the restrictive measures implemented by guardians. The latter need to produce consent, *i.e*., modify wards’ emotions, which adds to the emotional burden. Moreover, while they are recommended to *care,* they are also taught to refrain from *caring “too much,” i.e.,* giving too much time and attention. This makes it necessary to distance themselves from the sadness of wards’ situations. At the same time, showing a “stoic” expression when imposing a legal measure can be seen as morally dubious and “uncaring” (Rivera 2014). This difficult balance between adopting a caring attitude and a firmer one requires significant emotional labor, which is accentuated by the situation of forced assistance, which necessitates the recurrent use of coercion. In sum, social and moral taints *exacerbate* emotional labor, making it *dirty*. They endanger the respectability of professionals who must take care of stigmatized people while simultaneously putting them at risk of being presumed heartless when applying coercive measures.

### Variability in Taints Management

Similarly to what Hodson (2001) has demonstrated about other occupations, legal guardians derive a sense of achievement from three components: *autonomy* in organizing their work, strong and supportive *relationships* with colleagues, and the *variety of their tasks*. However, this paper shows that more is needed to fully address the “job satisfaction paradox,” *i.e.,* the fact that workers derive satisfaction or pride from performing a profession that cumulates strong constraints and is commonly perceived as inherently unsatisfying (Deery et al. 2019). We argue that in parallel with collective resources or strategies, we need to look at individual variations in taint management conducts. Mapping divergent tastes and distastes casts light, in a nuanced way, on the reasons why some can find certain tasks gratifying while others dislike them. It shows that hardship or tainted dimensions are not perceived uniformly and univocally.

The varying sensitivity of professionals to distinct categories of taints is rarely considered in the sociology dedicated to stigmatized jobs. A few previous studies have shown variations regarding taints management, for example among garbage workers (Bouville et al. 2022). Although insightful, these studies often concern the physical and social taints, which are presented as indissociable. We show that systematic correspondences exist between profiles, tastes, and taint management practices. Establishing profiles allows for a more constructivist and nuanced understanding of the social conditions under which specific taints affect workers, and of what mitigates their impact on job satisfaction.

Workers closed to the “compassionate pole” prefer social tasks to administrative or legal ones and are more often “sympathetic” than “apathetic” (Seim 2020, 68–81). They display no aversion to malodors or physical detritus in the domestic environment; instead, they regard home visits as a positive experience and an opportunity to spend quality time in the company of the wards. These legal guardians build justifications allowing them to stay away from the *moral taint*, as the suspicion they most fear is one of abusing or overusing their power. As a result, they tend to reframe their work as a form of social work directed toward a vulnerable and abandoned population, from which they derive a sense of pride in helping those left behind – an outlook that fuels and legitimizes rescuer dispositions and, at times, a “savior” identity. This self-understanding is sustained through everyday practices and professional discourses that emphasize care, protection, guidance, and moral responsibility rather than control or constraint. They routinely euphemize the coercive aspects of their decisions by framing them as acts of support, prevention, or benevolent intervention, and present their role as one of accompaniment, listening, and emotional availability. Emotional labor thus becomes central and is embraced as an engaging and fulfilling dimension of work. These professionals display well-developed skills in regulating their own emotions, cultivating empathy and patience, and actively working on the emotions of the wards – such as calming anger, fostering trust, or encouraging gratitude.

By contrast, workers closer to the “legal-rational” pole fear more the *social taint* associated with being in contact with an undeserving population. Consequently, they reframe their role as regulatory and firm. They do not put the relational aspects of their work at the center of their job definition. Their taste for administrative challenges makes them avoid the relational tasks that seem most (physically and socially) contaminating. Their interest lies in their curiosity and even fascination with deviant behaviors. They draw on the confrontation with the “underside of life” (Perry 1998, 113) to develop a feeling of singularity and even pride. Because the behaviors of wards often challenge their worldviews, legal guardians with this profile tend to put on hold some of their dispositions to avoid a lack of “patience” and cope with the lack of merit they can attribute to wards. To achieve this, they need to consider wards as not responsible for their behaviors. As observed in the law enforcement professions (see Literature Review), these legal guardians refer to the legal framework to alleviate the dubious nature of coercion and delegate the moral responsibility associated with the use of control. In interviews, they exhibit a proclivity for coercive means and demonstrate no reservations about engaging in conflict. They tend to label their ability to cope with complicated situations as heroic.

### Biographical Analysis: How the Past of Workers Shapes Taints Management

We rely on the assumption that *past* experiences of workers shape their *present* practices and attitudes at work (Pichonnaz 2021). This biographical approach makes it possible to show that gender and class socialization, as well as social mobility patterns, affect the ways in which workers deal with the tainted aspects of their job.

#### Gender and Styles of Femininity

A substantial body of research conducted in male-dominated occupations has found that workers derive satisfaction and exhibit pride by drawing on masculine attributes reinforced by the nature of the job, including working outdoors, using strength, showing physical resistance, and not being afraid of inherent risks (Ackroyd and Crowdy 1990; Bret 2020; Meara 1974; Perry 1998; Thompson, W. 1991). This paper addresses the ways workers in a position of authority, mainly women, deal with care. In this profession, authority is manifested through decisions made without wards’ consent and through the allocation of economic resources. Our study looks at the attempts to conform to hegemonic masculinity and distinguishes between different styles of femininity. While their shared professional ethos includes coercion, administrative work, *and* care, workers put an emphasis on varying components, depending on their gender or style of femininity. Some female guardians draw on an ethic of care to reframe the undesired population they help as “abandoned.” They undermine their authority and insist on the fact that their use of coercion remains consistent with the interests of the wards. Other women show a taste for conflict and coercion, and display skills diverging from the common representations of female occupations. These guardians, like Carine and Eloise, are more inclined to use coercion and less prone to show compassion than women with an ethic of care. They are therefore closer to middle-class masculine values (discipline, rationality, emotional distance) and masculine modalities of reframing the moral taint. Their taint management is more in line with that of male workers exhibiting hegemonic masculinity scripts (see the example of Greg), associated with rationality, toughness, power, control, assertiveness, or restricted emotions (Canham 2009; Schofield et al. 2000). This distinction between styles of femininity resonates with work on policing, in particular that of Susan E. Martin (1980) who distinguishes two profiles of female officers: police*women* (who emphasize their identity as women in a traditionally male profession) and *police*women (who adopt a more masculine, crime-fighting identity to fit into the police culture). We show that this distinction is rooted in the gender socializations of professionals, which has prevented “legal-rational” women from developing an ethic of care and have led them, at least in the case of Carine, to identify with the masculine authority figure of a father. Care is linked to socially and culturally constructed gender roles (Gilligan 1977). The lifelong assignment of people to caring tasks (Glenn 2010) may build specific aspirations and attitudes in which helping and caring for others become central concerns, shaping how women from the compassionate pole deal with their job taints.

#### Family Socialization, Class Habitus and Merit Attribution

If we look at the effect of class socialization, it must be noted that wards frequently exhibit behaviors that are incongruent with dominant definitions of merit. They often encounter difficulties in entering the labor market and may engage in drug use. All professionals cited in this article have pursued higher education and are employed in a middle-class profession. They come from middle-class families, occasionally upper-middle-class ones. Consequently, it is not surprising that some of them experience difficulty providing assistance to those they can see as undeserving, particularly when these individuals exhibit a reluctance to accept help. This discrepancy can be examined through the lens of class relations, particularly focusing on the values attributed to work and not “letting oneself go,” to individual responsibility, and to merit associated with effort that are strongly associated with the “class habitus” (Bourdieu 1991, 83) of middle classes (Bourdieu 1984). We have shown that, for professionals closest to the legal-rational pole, supporting certain people requires them to put these judgments, these class dispositions, on hold, which would otherwise lead them to attribute insufficient merit to them. To do this, professionals try to shift responsibility for their actions away from the wards. This phenomenon is not observed among professionals closer to the compassionate pole, even though they come from the same social classes as their colleagues. We explain this phenomenon by the compassionate dispositions internalized by these workers, which make it easier for them to attribute merit to wards. Taking responsibility for the fate of the wards, as opposed to the delegation of responsibility for their circumstances to them, is indeed indicative of an ethic of care. Socialization to care seems to weaken the internalization of the patterns of merit attribution usually identified in the middle classes.

#### Social Mobility Patterns and Unfulfilled Aspirations

The class structure is expressed in the workplace in another way, stemming from aspirations and social mobility patterns. The fact that professionals close to the legal-rational pole are either in a position of unfulfilled aspirations for success (Greg) or aspire to work in another sector (law enforcement for Carine or prisons for Eloise, for example), while those with a compassionate approach talk about their “vocation” for social work in general, and adult protection in particular, indicates that occupying a position that disappoints initial aspirations, which themselves depend on the class of origin, has an impact on work. Professionals with unfulfilled aspirations tend to distance themselves from the social work field, seeking legitimacy within the legal professions or, more fundamentally, adapting their work to align more closely with their professional aspirations, by emphasizing regulatory aspects and coercion. Recasting their profession as closely linked to the “legal order” allows them to both take a symbolically meaningful position (Emirbayer and Johnson 2008; Simpson et al. 2014) and compensate for the moral and social taints associated with their work. Here, it is not the internalization of merit attribution schemes that is at stake, but aspirations for social success, what Bourdieu calls “subjective expectations,” which are contingent on “objective chances” (Bourdieu 2000, 216) offered by the class position of workers’ family of origin.

## Footnotes

1 Depending on national contexts, these workers are labelled “professional curators,” “legal guardians,” “conservators,” or “trustees.” In this article, we refer to them as “(professional) legal guardians,” which is the most neutral terminology in our eyes. Alex V. Barnard (2023) has produced one of the few existing studies on guardianship. Drawing on rich empirical material, he offers a critical analysis of the ways in which the Californian State and its network of institutions and professionals engage with people living with severe mental health problems, and he documents the shortcomings of the guardianship system.
